# A nationally representative study on socio-demographic and geographic correlates, and trends in tobacco use in Nepal

**DOI:** 10.1038/s41598-019-39635-y

**Published:** 2019-02-25

**Authors:** Nipun Shrestha, Suresh Mehata, Pranil Man Singh Pradhan, Deepak Joshi, Shiva Raj Mishra

**Affiliations:** 1 Institute for Health and Sport (IHES), Victoria University, Melbourne, Australia; 2 Ipas Nepal, Kathmandu, Nepal; 3 Department of Community Medicine and Public Health, Institute of Medicine, Tribhuvan University, Kathmandu, Nepal; 4 Health and Nutrition Department, Save the Children, Kathmandu, Nepal; 5 Nepal Development Society, Chitwan, Nepal

## Abstract

Tobacco control still poses an immense challenge for the government of Nepal. Updated knowledge on the current pattern of tobacco use and its associated factors will be helpful for policy makers to curb the tobacco epidemic. This study fills this gap by, (i) exploring demographic, socio-economic and geographic correlates of current tobacco use using a nationally representative sample of 15–49-year adults from Nepal Demographic Health survey 2016, and (ii) examining the prevalence and trends of both smoking and non-smoking forms of tobacco use in a nationally representative sample of 15–49-year adults drawn from three consecutive Demographic Health Surveys (DHS) between 2006 and 2016.Among males, the prevalence of smokeless tobacco use was higher than that of smoking (40.1% and 27.4% respectively), whereas among females smoking was more common than smokeless tobacco use (prevalence of 5.5% and 3.8% respectively). Both smoking and smokeless tobacco use were associated with older age and lower level of education. Among males, those living in urban areas were more likely to consume any form of tobacco. Residents of terai/plains were more likely to use smokeless tobacco. The concentration curves on cumulative proportion of tobacco use ranked by wealth quintiles showed tobacco use to be highest among the lowest socio-economic groups in both males and females in all three survey years. We found a decreasing trend of tobacco smoking and an increasing trend of smokeless tobacco use over the 10-year period. However, the consumption of both forms of tobacco increased in young males during the same period. Proper monitoring of adherence to directives of the anti-tobacco law should be ensured to curb the increasing burden of tobacco use among young males, and a similar effort is needed to sustain the decline in tobacco uses among other population groups in Nepal.

## Introduction

Tobacco use has been attributed to be one of the leading causes for the growing burden of non-communicable diseases^1,2^. In 2015, consumption of tobacco products accounted for 11.5% of global deaths and 6% of global disability-adjusted life years (DALYs)^2^. Most of these deaths (~80%) occur in low middle-income countries where access to health care services are limited^3,4^. To combat the tobacco epidemic, the WHO Framework Convention on Tobacco Control (WHO FCTC) treaty was introduced in 2003^5^. Being a signatory of the treaty, the government of Nepal assented to the anti-tobacco directives of Tobacco Product Control and Regulatory Act 2010 in November, 2011^6,7^. This was regarded as a landmark in Nepal’s campaign against tobacco. Since then, Nepal has made significant progress on tobacco control. This has been reflected strongly in the current policy which enforces smoke-free healthcare facilities, educational institutions, government facilities, restaurants, offices and public transports. Although Nepal is yet to implement plain packaging, the percentage of the pack covered with a large health warning (90%) is the highest among the countries of this region^8^.

Despite all the efforts, tobacco use continues to be a significant burden among the Nepalese people. More than 27100 Nepalese people are killed by tobacco-caused diseases every year and more than 21000 children (10–14 years old) and 3745000 adults (15+ years old) continue to consume tobacco every day^8^.

The major form of tobacco use in Nepal is cigarette smoking along with hand rolled cigarettes (bidis). Various form of smokeless tobacco (SLT) are also consumed like gutkha (contains areca nut, tobacco, catechu and sweet flavor), zardapaan or betel quid (rolled betel leaf with lime, betel nut and tobacco), khaini (flavored tobacco mixed with lime), and sokha (non-flavored raw leaf of tobacco crushed manually, mixed with lime and rolled in hands before use)^9,10^. SLT has been found to be associated with many oral diseases along with cancer of head and neck, oesophagus and pancreas^11,12^.

Nepal has undergone a significant political change over the last decade. After the people’s movement in 2006, the socio-political landscape has changed considerably also affecting public policy and governance. The implementation of graphic health warning (GHW) brought by the government then in 2011 is considered as a milestone in Nepal, and is clearly an example which lot of other countries can follow. Previous studies have revealed that people with less education, poor economic conditions and older age groups were more likely to consume tobacco in Nepal^9,10^. However, there is a lack of information on prevalence estimates in tobacco use and its distribution in Nepal, and temporal trends in its use based on nationally representative data. Updated knowledge on current pattern of tobacco use and trends in prevalence of tobacco use will be beneficial for policy makers to curb the current tobacco epidemic^9^. The aim of this study was to examine trends in the prevalence of tobacco use from 2006 to 2016 and to explore associations of tobacco use with demographic, socio-economic and geographic variables in 2016 in a nationally representative sample of 15–49-year adults in Nepal.

## Results

### Demographic and socioeconomic correlates of tobacco use in 2016

The overall prevalence rate for any form of tobacco use was higher in males (52.3%) than in females (8.4%). Similar differences were also reported for tobacco smoking (27.4% in males and 5.5% in females) and SLT use (40.1% in males and 3.8% in females). The unadjusted analysis showed that young males and females below 25 years had the lowest prevalence of tobacco use (Table 1). These findings correspond with adjusted analysis which found age to be a significant predictor of tobacco use. Compared to adolescent males and females aged 15–19 years, other age groups had higher adjusted odds of using all forms of tobacco (Table 2). Similarly, in unadjusted analysis males and females with no formal education had the highest prevalence of any form of tobacco use (77% for male and 18% for females) (Table 1). The adjusted analysis also found lower educational status to be significantly associated with the use of any form of tobacco. Males with no formal education were 2.86 times (CI 1.92–4.17) more likely to smoke tobacco than males with higher education. Conversely, females with no formal education were 6.67 times (CI 2.38–20) more likely to smoke tobacco than their counterparts with higher education (Table 2).

**Table 1:** Prevalence of tobacco use among Nepalese males and females aged 15–49 years by socio-demographic characteristics, Nepal Demographic and Health Survey 2016 (N = 4063 males and 12862 females).

	Male				Female			
	Total participants	Current smoking	Current use of non smoking tobacco	Any form of tobacco	Total participants	Current smoking	Current use of non smoking tobacco	Any form of tobacco
	n	n(%)	n(%)	n(%)	n	n(%)	n(%)	n(%)
**Age (years)**								
15–19	931	147 (15.8)	154 (16.5)	248 (26.6)	2598	13 (0.5)	10 (0.4)	22 (0.8)
20–24	649	203 (31.3)	198 (30.5)	314 (48.4)	2251	26 (1.2)	23 (1)	48 (2.1)
25–29	525	165 (31.4)	222 (42.2)	289 (55.1)	2135	51 (2.4)	57 (2.7)	91 (4.3)
30–34	535	165 (30.8)	280 (52.5)	346 (64.8)	1806	97 (5.3)	79 (4.4)	162 (9)
35–39	544	146 (26.8)	295 (54.1)	338 (62.1)	1572	122 (7.7)	113 (7.2)	211 (13.4)
40–44	463	133 (28.7)	261 (56.4)	305 (65.9)	1388	194 (14)	114 (8.2)	276 (19.9)
45–49	415	153 (36.9)	221 (53.1)	286 (68.9)	1113	207 (18.6)	96 (8.6)	272 (24.4)
**Educational level**								
No education	391	149 (38.1)	254 (64.9)	303 (77.4)	4281	555 (13)	312 (7.3)	771 (18)
Primary	789	290 (36.7)	467 (59.2)	558 (70.8)	2150	100 (4.7)	120 (5.6)	201 (9.4)
Secondary	1990	503 (25.3)	730 (36.7)	969 (48.7)	4516	43 (1)	54 (1.2)	94 (2.1)
Higher	894	170 (19.1)	180 (20.1)	297 (33.2)	1915	11 (0.6)	6 (0.3)	15 (0.8)
**Place of residence**								
Urban	2647	757 (28.6)	1007 (38)	1364 (51.5)	8072	415 (5.1)	268 (3.3)	617 (7.6)
Rural	1416	356 (25.1)	623 (44)	763 (53.9)	4790	294 (6.1)	223 (4.7)	465 (9.7)
**Province**								
Province 1	691	186 (26.9)	285 (41.2)	371 (53.7)	2173	69 (3.2)	166 (7.6)	223 (10.3)
Province 2	795	174 (21.8)	465 (58.5)	494 (62.2)	2563	52 (2)	33 (1.3)	73 (2.9)
Province 3	1009	324 (32.1)	242 (24)	452 (44.8)	2732	212 (7.8)	62 (2.3)	245 (9)
Province 4	376	92 (24.6)	107 (28.4)	158 (42)	1249	78 (6.2)	71 (5.7)	132 (10.6)
Province 5	658	145 (22)	324 (49.2)	364 (55.2)	2274	93 (4.1)	79 (3.5)	153 (6.7)
Province 6	203	59 (29)	72 (35.4)	97 (47.9)	724	95 (13.1)	31 (4.3)	114 (15.7)
Province 7	330	132 (40.2)	136 (41.2)	190 (57.6)	1145	111 (9.6)	50 (4.3)	141 (12.4)
**Ecological zone**								
Mountain	252	81 (31.9)	72 (28.4)	119 (47.2)	775	73 (9.4)	47 (6.1)	110 (14.1)
Hill	1791	502 (28)	534 (29.8)	812 (45.3)	5556	413 (7.4)	254 (4.6)	598 (10.8)
Terai	2019	530 (26.2)	1025 (50.7)	1196 (59.2)	6531	223 (3.4)	191 (2.9)	374 (5.7)
**Wealth quintile**								
Poorest	623	213 (34.2)	264 (42.3)	358 (57.4)	2176	281 (12.9)	179 (8.2)	411 (18.9)
Poorer	706	199 (28.2)	330 (46.8)	404 (57.2)	2525	167 (6.6)	135 (5.3)	269 (10.6)
Middle	758	208 (27.4)	362 (47.8)	438 (57.8)	2595	99 (3.8)	78 (3)	163 (6.3)
Richer	982	278 (28.3)	435 (44.3)	530 (54)	2765	115 (4.2)	63 (2.3)	163 (5.9)
Richest	994	215 (21.6)	239 (24.1)	397 (39.9)	2801	47 (1.7)	37 (1.3)	76 (2.7)
**Frequency of reading newspaper or magazine**								
Not at all	1822	516 (28.3)	876 (48.1)	1065 (58.4)	8950	662 (7.4)	447 (5)	994 (11.1)
Less than once a week	1360	372 (27.4)	522 (38.4)	692 (50.9)	2793	38 (1.4)	36 (1.3)	71 (2.5)
At least once a week	881	225 (25.5)	232 (26.4)	370 (42)	1119	9 (0.8)	8 (0.7)	17 (1.5)
**Frequency of listening to radio**								
Not at all	1154	320 (27.7)	487 (42.2)	645 (55.9)	5558	337 (6.1)	274 (4.9)	544 (9.8)
Less than once a week	1441	397 (27.6)	587 (40.8)	764 (53)	3738	242 (6.5)	121 (3.2)	328 (8.8)
At least once a week	1468	396 (27)	557 (37.9)	717 (48.8)	3566	130 (3.7)	97 (2.7)	210 (5.9)
**Frequency of watching television**								
Not at all	806	238 (29.5)	393 (48.8)	480 (59.6)	3700	337 (9.1)	227 (6.1)	499 (13.5)
Less than once a week	1185	344 (29)	495 (41.8)	629 (53.1)	2692	185 (6.9)	109 (4.1)	262 (9.7)
At least once a week	2073	531 (25.6)	743 (35.8)	1018 (49.1)	6470	187 (2.9)	156 (2.4)	320 (5)
**Use of internet**								
Never	2028	635 (31.3)	1058 (52.1)	1280 (63.1)	9782	676 (6.9)	463 (4.7)	1021 (10.4)
Yes, within 12 months	1914	442 (23.1)	522 (27.3)	780 (40.7)	2970	33 (1.1)	29 (1)	60 (2)
Yes, but not in the last 12 months	121	35 (29.1)	51 (42.2)	67 (55.3)	111	0	0	0
Total	4063	1113 (27.4)	1630 (40.1)	2127 (52.3)	12862	709 (5.5)	492 (3.8)	1082 (8.4)

**Table 2:** Correlates of tobacco smoking, smokeless tobacco use and any tobacco use.

	Male						Female					
	Smoking		Smokeless tobacco		Any form		Smoking		Smokeless tobacco		Any form	
	AOR	95% CI	AOR	95% CI	AOR	95% CI	AOR	95% CI	AOR	95% CI	AOR	95% CI
**Age (years)**												
15–19	1		1		1		1		1		1	
20–24	2.69*	2.04–3.56	3.04*	2.21–4.17	3.39*	2.63–4.37	1.94	0.75–4.96	2.38*	1.19–4.76	2.26*	1.20–4.27
25–29	2.58*	1.83–3.63	5.18*	3.67–7.32	4.25*	3.16–5.72	2.80*	1.26–6.20	5.17*	2.55–10.48	3.47*	1.98–6.11
30–34	2.18*	1.58–3.02	7.03*	4.97–9.93	5.55*	4.08–7.53	5.29*	2.68–10.42	7.28*	3.58–14.81	6.57*	3.86–11.19
35–39	1.64*	1.20–2.25	7.33*	5.41–9.93	4.50*	3.29–6.14	7.25*	3.58–14.68	10.79*	5.54–21.03	9.47*	5.63–15.92
40–44	1.90*	1.34–2.69	7.77*	5.68–10.62	5.41*	3.95–7.41	12.55*	6.15–25.63	11.78*	5.76–24.08	14.30*	8.24–24.83
45–49	2.77*	1.95–3.94	6.30*	4.40–9.03	5.88*	4.26–8.13	17.20*	8.70–34.01	11.86*	5.66–24.86	17.76*	10.37–30.41
**Education level**												
No education	1		1		1		1		1		1	
Primary	0.90	0.67–1.21	1.33	0.94–1.87	1.05	0.73–1.51	0.55*	0.39–0.77	0.94	0.75–1.19	0.69*	0.56–0.84
Secondary	0.58*	0.43–0.80	0.80	0.56–1.14	0.60*	0.43–0.86	0.22*	0.15–0.33	0.39*	0.26–0.60	0.28*	0.21–0.39
Higher	0.35*	0.24–0.52	0.39*	0.26–0.58	0.31*	0.21–0.47	0.15*	0.05–0.42	0.14*	0.05–0.39	0.13*	0.06–0.28
**Place of residence**												
Urban	1		1		1		1		1		1	
Rural	0.78*	0.63–0.96	0.80*	0.66–0.98	0.79*	0.66–0.95	0.87	0.68–1.10	1.06	0.80–1.40	0.96	0.78–1.17
**Province**												
Province 1	1				1		1		1		1	
province 2	0.97	0.69–1.38	1.33	0.91–1.96	1.05	0.74–1.48	0.83	0.41–1.69	0.26*	0.14–0.50	0.36*	0.22–0.61
province 3	1.59*	1.08–2.36	0.60*	0.40–0.90	0.97	0.70–1.33	3.25*	2.09–5.06	0.29*	0.16–0.51	0.94	0.66–1.34
province 4	1.02	0.69–1.51	0.69*	0.49–0.98	0.76	0.53–1.10	1.90*	1.16–3.11	0.58*	0.40–0.84	0.85	0.61–1.19
province 5	0.75	0.56–1.00	1.14	0.85–1.52	0.88	0.67–1.16	1.38	0.88–2.18	0.49*	0.33–0.73	0.66*	0.48–0.91
province 6	1.08	0.70–1.66	0.85	0.57–1.28	0.84	0.57–1.24	3.02*	1.97–4.64	0.31*	0.19–0.52	0.96	0.70–1.32
province 7	1.65*	1.18–2.32	1.01	0.70–1.47	1.21	0.88–1.65	2.67*	1.70–4.19	0.46*	0.30–0.71	0.98	0.69–1.38
**Ecological zone**												
Mountain	1		1		1		1		1		1	
Hill	0.93	0.64–1.35	1.63*	1.24–2.13	1.23	0.96–1.59	1.15	0.76–1.74	0.94	0.62–1.44	1.05	0.74–1.48
Terai	1.13	0.71–1.81	1.93*	1.20–3.08	1.67*	1.18–2.37	1.34	0.80–2.24	1.35	0.81–2.23	1.43	0.91–2.25
**Wealth quintile**												
Poorest	1		1		1		1		1		1	
Poorer	0.82	0.62–1.09	0.93	0.68–1.26	0.84	0.66–1.07	0.75	0.54–1.04	0.83	0.58–1.19	0.74*	0.56–0.99
Middle	0.89	0.62–1.27	0.73	0.50–1.07	0.75	0.55–1.01	0.57*	0.39–0.83	0.54*	0.34–0.85	0.52*	0.37–0.73
Richer	0.89	0.61–1.30	0.83	0.54–1.26	0.72*	0.52–0.99	0.57*	0.36–0.91	0.41*	0.22–0.77	0.45*	0.29–0.71
Richest	0.60*	0.40–0.89	0.46*	0.30–0.71	0.50*	0.36–0.71	0.27*	0.13–0.56	0.32*	0.18–0.59	0.25*	0.14–0.42
**Frequency of reading newspaper or magazine**												
Not at all	1		1		1		1		1		1	
Less than once a week	1.09	0.87–1.36	0.95	0.76–1.18	1.03	0.81–1.29	0.81	0.50–1.32	0.81	0.55–1.20	0.82	0.57–1.19
At least once a week	1.23	0.85–1.78	0.88	0.66–1.18	1.03	0.76–1.41	0.76	0.28–2.08	0.83	0.35–2.01	0.86	0.44–1.67
**Frequency of listening to radio**												
Not at all	1		1		1		1		1		1	
Less than once a week	0.86	0.69–1.07	1.02	0.82–1.26	0.85	0.69–1.06	1.01	0.83–1.23	0.58*	0.44–0.76	0.80*	0.67–0.95
At least once a week	0.81	0.65–1.01	1.03	0.78–1.34	0.75*	0.61–0.92	0.76	0.57–1.01	0.61*	0.45–0.83	0.69*	0.54–0.88
**Frequency of watching television**												
Not at all	1		1		1		1		1		1	
Less than once a week	1.01	0.75–1.37	1.06	0.79–1.42	1.03	0.77–1.39	0.97	0.71–1.34	0.99	0.71–1.4	0.99	0.77–1.27
At least once a week	1.01	0.78–1.31	1.29	0.97–1.72	1.29	0.96–1.74	0.78	0.53–1.13	1.07	0.76–1.51	0.94	0.71–1.24
**Internet use**												
Never	1		1		1		1		1		1	
Within 12 months	0.95	0.77–1.17	0.83	0.68–1.01	0.86	0.70–1.06	1.41	0.79–2.51	0.97	0.59–1.61	1.30	0.88–1.92
Yes, but not in the last 12 months	0.98	0.58–1.67	0.81	0.49–1.33	0.88	0.54–1.42						

In unadjusted analyses, the prevalence of tobacco smoking (34.2%) was highest for the men from the poorest households whereas the women from poorest households had highest prevalence for tobacco smoking (12.9%) as well as smokeless tobacco use (8.2%) and any forms of tobacco use (18.9%). After adjustment, odds of any tobacco use decreased with increasing wealth for both men and women, although the trend appears to be more marked for women. For instance, women from poorest households were four times (CI 2.38–7.14) more likely to use any form of tobacco than women from richest households (Table 2). Similarly men from poorest households were two times (CI 1.41–2.78) more likely to use any form of tobacco than men richest households. Media exposure particularly television, internet and newspaper/magazine showed no significant relationship with any kind of tobacco use. However, women who listened to the radio at least once a week had lower odds (AOR 0.69, CI 0.54–0.88) of using SLT compared to women who did not listen to the radio (Table 2).

### Geographic correlates of tobacco use in 2016

In unadjusted analysis, prevalence of any form of tobacco use among males was 51.5% in urban areas and 53.9% in rural areas. Similarly, prevalence of any form of tobacco use among females was 7.6% in urban areas and 9.7% in rural areas (Table 1). Multivariable analysis showed that males from urban areas had lower odds of using any form of tobacco, including both smoking (AOR 0.78, CI 0.63–0.96) and SLT (AOR 0.80, CI 0.66–0.98) than their counterparts from rural areas. On the contrary, females showed no such significant association of urban-rural residence with tobacco use (Table 2).

**Figure 1:**
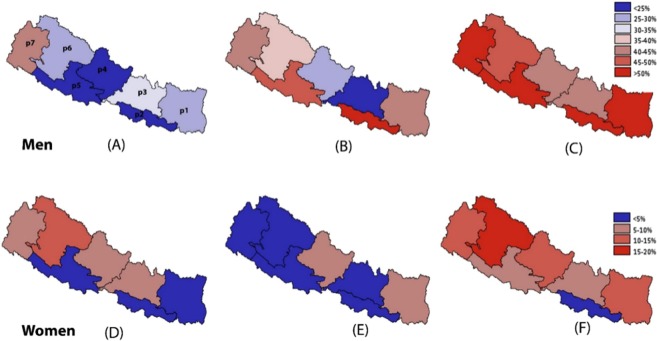
Geographic distribution of Tobacco use (**A**,**D**: current smoking, **B**,**E**: any non-smoking form of tobacco, **C**,**F**: any forms of tobacco) by seven provinces of Nepal [p1: province-1 (Eastern mountain, hills and terai), p2: province-2 (Eastern terai), p3: province-3 (Central mountains, hills and terai), p4: province-4 (Western mountain, hills and terai), p5: province-5 (Western mountain, hills and terai), p6: province-6 (Far-western mountain and hills), p7: province-7 (Far-western mountain, hills and terai)]. Legends are the cluster adjusted prevalence (%) for the particular province.

In unadjusted analysis, prevalence of tobacco smoking was highest among males in Province 7 (40.2%) and highest among females in Province 6 (13.1%; Table 1, Fig. 1). But after adjustment, males in Province 3 (AOR 1.59, CI 1.08–2.36) and Province 7 (AOR 1.65, CI 1.18–2.32) were more likely to smoke tobacco than males in Province 1 (most advantaged in political and economic means). Similarly females in Province 3 (AOR 3.25, CI 2.09–5.06), Province 6 (AOR 3.02, CI 1.97–4.64) and Province 7 (AOR 2.67, CI 1.70–4.19) were more likely to smoke tobacco than females in Province 1 (Table 2). In unadjusted analysis, SLT use was the highest among males in Province 2 (58.5%) and among females in Province 1 (7.6%). After adjustment, there was no significant difference in the likelihood of SLT consumption between males in Province 2 and Province 1 but females in province 1 had highest likelihood of SLT use compared to all other Provinces. The adjusted analysis also showed males in Province 3 (AOR 0.60, CI 0.40–0.90) and Province 4 (AOR 0.69, CI 0.49–0.98) were less likely to consume SLT than males in Province 1. Women in Province 2 (AOR 0.26, CI 0.14–0.50), Province 3 (AOR 0.29, CI 0.16–0.51) and Province 6 (AOR 0.31, CI (0.19–0.52) were less likely to consume SLT than women in Province 1 (Table 2). The highest prevalence of any form of tobacco use was reported from Province 2 (62.2%) for males and from Province 6 (16.7%) for females (Table 1, Fig. 1). In unadjusted analysis, the disparity was also observed according to the ecological region where the use of SLT among males were highest in Terai region (50.7%) compared to other ecological regions (<30%) (Table 1). In adjusted analysis, males from Terai region had nearly two times higher odds (AOR 1.93, CI 1.20–3.08) of SLT use compared to males from Mountainous region (Table 2).

### Trends in tobacco use in Nepal

**Figure 2:**
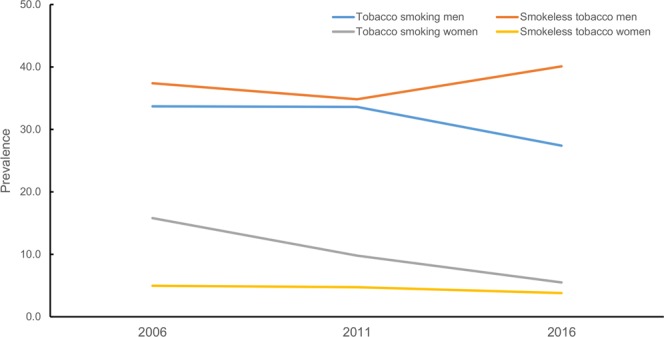
Trend of tobacco smoking and smokeless tobacco use among Nepalese males and females aged 15–49 years.

**Figure 3:**
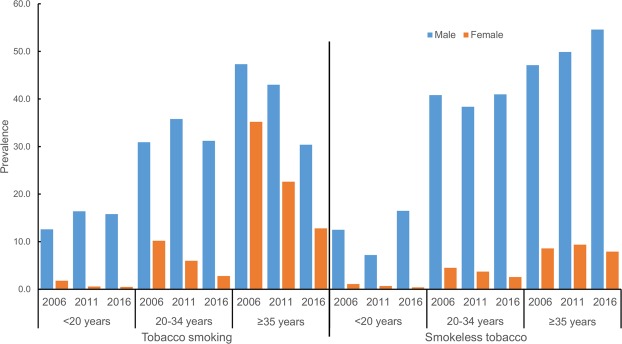
Trend of tobacco smoking and smokeless tobacco use among Nepalese males and females 15–49 years disaggregated by age groups.

**Figure 4:**
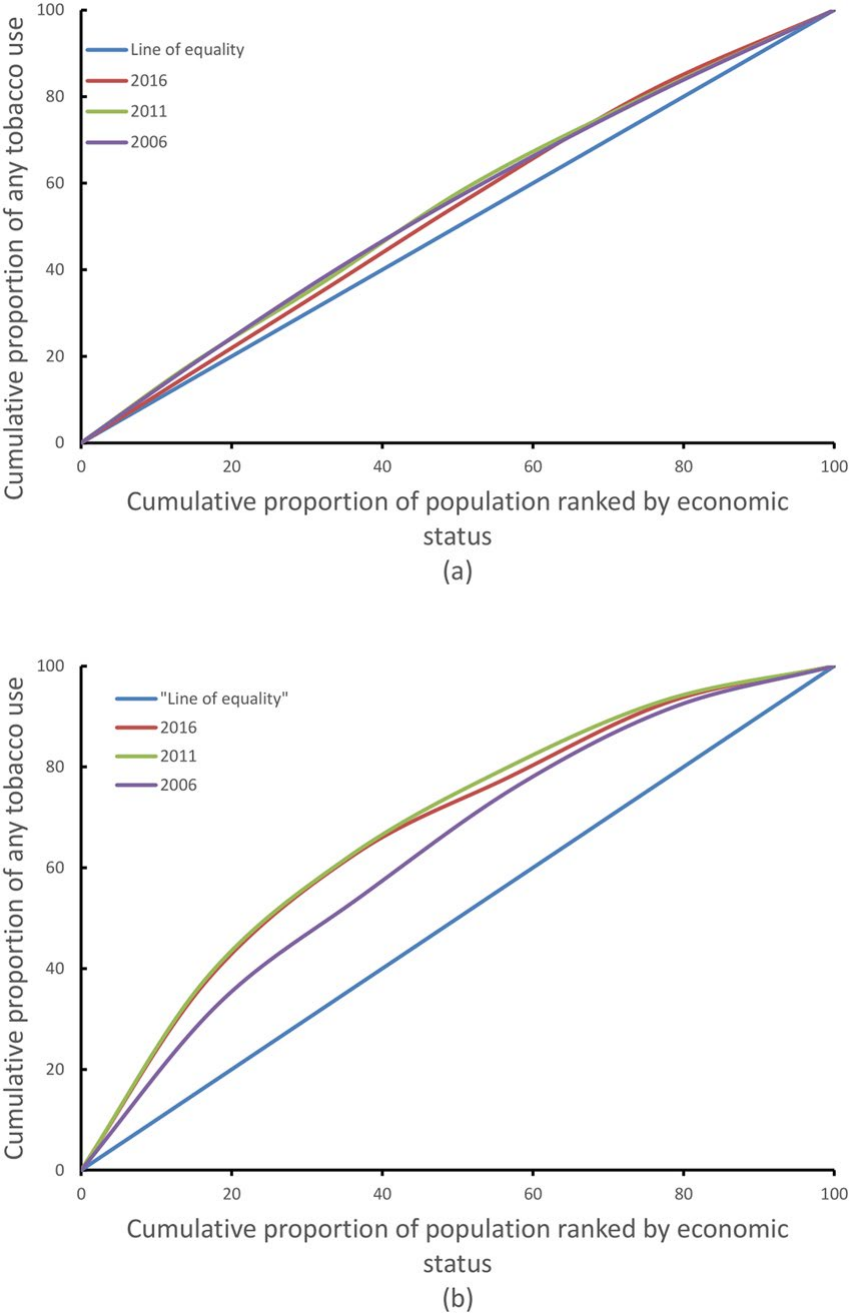
Concentration curve by any tobacco use among males (**a**), and females (**b**), Nepal Demographic Health Survey 2006–2016.

The overall prevalence of tobacco consumption has remained steady among males since 2006. However, the prevalence of tobacco smoking among males has declined whereas consumption of SLT has increased in the recent years (Fig. 2). There has been continuous decline in tobacco smoking and modest decline in SLT use among females over the last decade (Fig. 2). By age group, the prevalence of tobacco smoking declined most prominently among both males and females aged 35 and above. However, the prevalence of SLT increased in males aged 35 and above (Fig. 3). There was a negative gradient in tobacco uses across the wealth quintiles in both males and females (Fig. 4). In males, the bottom 20% constituted nearly 22% of tobacco consumers while the middle 60% constituted nearly 63%, and the top 20% constituted nearly 15% of tobacco users in 2016. (Fig. 4a). However the disparity was more evident in females, the bottom 20% constituted nearly 43% of tobacco users and the top 20% constituted nearly 7% of tobacco users in females (Fig. 4b). The adjusted analyses for 2016 also showed similar results with the odds ratios decreasing across quintiles for increasing wealth, both for men and women.

## Discussion

This sub-analysis of the data collected by a nationally representative survey (NDHS 2016) provided the current status of tobacco use and its distribution among adults aged 15–49 years in Nepal. The overall prevalence of tobacco use was 52.3% in males and 8.4% in females. Non Communicable Diseases (NCD) risk factors survey 2013 reported lower prevalence in males (48.1%) but higher prevalence in females (14.1%). This could be attributable to difference in the sampling design between these surveys or perhaps is a true change in prevalence since 2013^13^. Our results showed marked gender difference in tobacco consumption which has been similarly reported from other South Asian countries^14–16^. We found that the distribution of tobacco consumption in Nepal varied not only across the population subgroups but geographic regions as well. The increasing prevalence of tobacco smoking by age, similar to that reported in previous demographic health surveys 2006^17^ and 2011^18^, is a reminder of the historical burden of tobacco use in Nepal. Around 36.7% of adolescent males used to smoke tobacco products in Nepal in 2001^19^. These males could have continued smoking as evident from the similar prevalence of tobacco smoking (30.8%) in males aged 30–34 years in 2016. It shows the failure of aggressive tobacco control program targeting the younger generations. The higher prevalence of tobacco consumption among the lesser educated population subgroup regardless of age-groups accentuates the inequities in distribution of this important risk factor for chronic diseases. For example, males with no formal education were 2.86 times (CI 1.92–4.17) and females with no formal education were 6.67 times (CI 2.38–20) more likely to smoke tobacco than those with higher education. The higher burden of tobacco consumption among lesser educated population was also seen in previous demographic health surveys^9,10^.

Tobacco use was highest amongst the lowest socio-economic groups in both males and females. However, the disparity in burden of tobacco consumption between rich and poor is narrowing in males whereas widening in females since 2006. The increase in tobacco taxes might have compelled the poor to quit tobacco, contributing to reduction in disparity in tobacco use among males in these recent years. The aftermath of people’s movement in 2006 brought a paradigm shift in socio-political sector giving women more autonomy and an equal status as men in the social and political sector^20^. This gender empowerment along with erosion of cultural and economic constraints might have favored tobacco use among women from low socioeconomic groups^21^. However the change in socio economic inequities over time might be different from that depicted by concentration curves because the concentration curve analysis doesn’t adjust for other variables. The significant association observed between tobacco use and place of residence (urban/rural) for males might be due to the fact that social and peer pressure, is particularly high among young males from urban areas, and drives them to initiate tobacco use at early ages. After adjusting for other variables, the likelihood of tobacco smoking was highest amongstmales in Provinces 3 and 7 and amongst females in Provinces 3, 6 and 7.The high likelihood of tobacco smoking in males in province 7 corroborates with the highest likelihood of tobacco smoking reported in Far western region of Nepal in previous DHS^9^. However, the high likelihood of tobacco smoking among males in Province 3 does not corroborate with likelihood of tobacco smoking among males in central region reported in previous DHS^9^. The adjusted analysis is however not available for females from previous demographic health surveys to make such comparisons. Similarly, highest prevalence of SLT use among men was reported in Province 2 (Eastern Terai).

Noticeably, the tobacco smoking prevalence has been increasing among adolescent males while declining among females of the same age group (Fig. 2). In Nepalese context, smoking is considered as an acceptable behavior for men but not for women. So, under-reporting of tobacco smoking in adolescent girls in urban areas is very likely. The increasing prevalence in tobacco smoking among adolescent males is driven by thrill seeking or risk-taking behavior in this age group. To put this other way round, an increasing prevalence highlights the weaknesses of existing tobacco control program in Nepal to reach the young masses who are critically vulnerable to the aggressive branding and corporate sponsorships of sports and musical events. Such strategy employed by tobacco companies to evade marketing restrictions by targeting alternative advertising avenues is of particular concern. Additionally, WHO’s Tobacco Assessment Report highlighted Nepal’s weakness in governing advertisements from international TV and radio stations^22^. Regarding corporate sponsorship, Nepal’s tobacco giant ‘Surya Nepal Pvt. Ltd.’, spent 14,617,994 Nepalese Rupees (approximately 146,179 USD) in 2016 on three core areas: promotion and sponsorship, donations and publishing books, and periodicals. Additionally, 17,781,540 Nepali rupees (approximately 177,815 USD) went into market research and a small total of 4,033,385 Nepali Rupees (approximately 40,333 USD) was spent in actual advertising^23^. The adaptation by tobacco industries to changed regulations can be seen with the aforementioned details of expenditures into market research, a higher denomination in sponsorship and a lower total in actual advertising. This will make the youth more vulnerable as the industries will seek respite on corporate sponsorships and improve public imagery of their brands through heavy donations into public and social-enterprises which the country has failed to monitor and govern^24^. For example, the government has been unable to fully implement graphic health warnings that cover 90% of the package of smoked and SLT products in Nepal. This regulation of 90% graphic health warnings is the largest in the region^25^.

The consumption of SLT (40.1%) is even higher than tobacco smoking (27.4%). The use of SLT is increasing among adolescent males, males aged 35 and above over the last decade (Fig. 2). This increasing trend might be due to shift in preference from tobacco smoking to SLT usage due to high taxes on smoking tobacco products and low taxation on SLT products^26^. There has been substantial growth in the import of chewing tobacco products (khaini, gutka, zarda) from India over the years in Nepal. In addition, Nepal has also seen a rampant growth of illegal production of SLT within the country in recent times^27^. However, there is a lack of comprehensive information on production, sales and revenue collection of SLT products in Nepal^24^. SLT use was found to be more prevalent in Terai areas closer to the border with India^28,29^. In contrast, residents of mountains and hills were more likely to be tobacco smokers. The higher burden of SLT use in Terai, and tobacco smoking in mountainous and hilly regions were also evident in previous demographic health surveys^9,10^. Hence, SLT control efforts need to be considered separately in order curb its increasing consumption.

In today’s society, the media plays a crucial role in shaping people’s understanding and attitude towards a certain issue. Exposure to mass media has been reported to cause increase in tobacco smoking^30^ and decline in SLT use^31^ in various studies. In this study, there was no significant association between television and internet use and likelihood of any forms of tobacco use. However, for radio use, frequent listeners were significantly less likely to consume any forms of tobacco, and particularly smokeless tobacco.

In Nepal, tobacco products can still be purchased in the shops and consumed even in public places^6,32^. This poses an immense challenge for the effective implementation of anti-tobacco law. We need further researches exploring the adherence to directives of the anti-tobacco law by the vendors especially outside the schools and colleges in Nepal. This would be the starting step in addressing the growing burden of tobacco use among the adolescents. Investing in tobacco control measures (tobacco tax increases, smoke-free policies, package warnings, and advertising bans, awareness campaigns) have proven to be cost effective in terms of saving health care costs and human productivity^33,34^. In addition, integrating tobacco cessation in routine primary health care settings also needs to be considered for those who desire to quit smoking^24^. Implementing these various tobacco control measures would require interdisciplinary and inter-sectoral collaborative endeavors among professionals and institutions that have traditionally worked separately.

### Graphic health warning (2011-present) and way forwards for plain packaging

**Figure 5:**
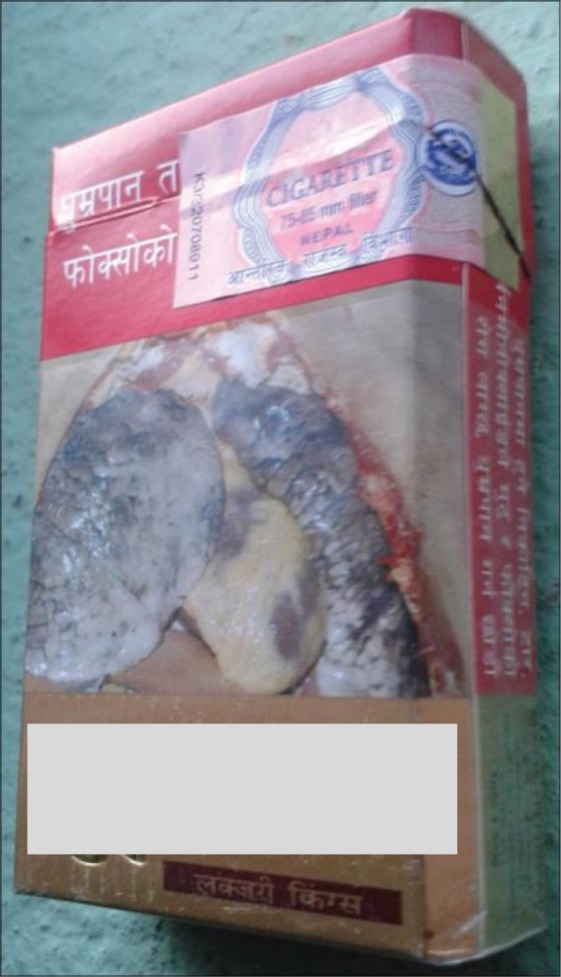
Health message in the new plain package has been subsided by VAT sticker. This image is reproduced from Mishra *et al*.^43^ with author’s permission.

Introduction of graphic health warning in tobacco products was started in 2011 with 75% coverage, and it was subsequently increased to 90% in 2014^35^. On the positive side again, the government of Nepal is planning to introduce plain packaging by the end of 2018. New tobacco products will not have any color, imagery, corporate logo and trademark^36^. However, there is a mismatch between the tobacco control efforts and monitoring of compliance of packaging rules. One such example is the use of sticker over the tobacco graphic warning (Fig. 5). Further, a proper monitoring of tobacco control in the changed scenario relied excessively on public system with no independent assessment and documentation. Detail examination on the effect of graphic warning and proposed plain packaging in tobacco use is necessary in future, especially among the young population in Nepal who are particularly vulnerable to the tobacco uses (any product). Consequently, the increasing tobacco smoking among adolescent males is a tale-tale sign of the relative inefficiency in the tobacco control program to target the young masses, and could also be due to better reporting of tobacco use in recent surveys.

Based on a large national sample that consists of both urban and rural populations in Nepal, findings of this study may be utilized for monitoring tobacco epidemic and assessing the prevalence among population subgroups at country-level. However, as in any cross-sectional study, we could not establish causal association between tobacco consumptions and its determinants. In a conservative society of Nepal, it is very likely that tobacco use based on self-report may be under-reported and needs to be verified by measuring urinary cotinine levels in future studies. We don’t have information on prevalence of tobacco use among those aged 50 years and over as NDHS has limited the age of males and females respondents from 15–49 years only. Though prevalence rates of tobacco consumption is declining among Nepalese women, very little is known about the prevalence and determinants of exposure to secondhand smoke from male smokers. This dimension of exposure to secondhand tobacco smoke needs to be considered in future demographic health surveys. Finally, our study informs the implementation of Tobacco Product Control and Regulatory Act 2010: reduction in SLT use and focus on adolescent males should become the priority for tobacco control efforts in Nepal.

## Conclusion

More than half of the Nepalese males and around 8% of Nepalese females aged 15–49 years were reported to have consumed tobacco. People from lower socioeconomic status and less education were more likely to consume tobacco. This study highlights a need for a greater vigilance on implementation of Tobacco Product Control and Regulatory Act 2010, targeting young males.

## Methods

### Data sources

We used data from a nationally representative Nepal Demographic Health Surveys carried out in 2006, 2011 and 2016 for this study. These surveys used a stratified two-stage cluster sampling in rural areas and three stages sampling in urban areas. Primary sampling units (wards) were selected with probability proportional to their size in the first step. Then, households for interviews were selected based on equal probability systematic selection criterion. A detailed study methodologies and description of survey variables for each survey year has been presented in respective DHS reports^17,18,37^. These surveys collected information about demographic factors, socio-economic factors and health status from all members aged 15 and above in the sample of households selected. Subsequently, all females aged 15–49 who stayed in the households the night before the survey were interviewed. In every alternate household selected, all males aged 15–49 years, who stayed in the households the night before the survey, were interviewed for tobacco using practices.

We investigated socio-demographic and geographic correlates only for 2016 as such analysis for 2006 and 2011 were already conducted in previously published papers^9,10^. All the three surveys were conducted using same methodology and collected information on the same variables.

### Variables

Information about tobacco use was obtained from the following questions that were asked in both male’s and females’s questionnaires of DHS:

1. Do you currently smoke tobacco every day, somedays, or not at all?
2. In the past, have you ever smoked tobacco every day, some days, or not at all?
3. Do you currently use smokeless tobacco every day, some days, or not at all?
4. On average, how many of the following products do you currently smoke? (Multiple response: Manufactured cigarettes/hand rolled cigarettes/pipes full of tobacco/cigars, cheroots or cigarillos/water pipe/any other).

For our analysis, dichotomous outcome variables were created from the information obtained from above questions. Any respondent answering yes to question 1, was categorised as “Tobacco smoker”. Similarly any respondent answering yes to question 3, was categorised as then “Smokeless tobacco user”. “Any form of tobacco user” in this study is defined as any person consuming cigarette/pipe/bidi/other smoking forms which were derived from the responses to question 1 and 3 above. If a respondent answers yes to either of question 1 or 3, then, he/she was categorised as any form of tobacco user.

#### Explanatory variable

We selected three categories of explanatory variables: socio-demographic, geographic and access to information. The socio- demographic variables were age of the participants, sex, educational status and wealth index. Educational status was grouped into four categories: no formal schooling, attended primary schooling, attended secondary schooling and higher (includes high school, college/university, and post graduate and above education). Wealth index was derived using principal component analysis which included information on number and kinds of consumer goods such as bicycle or car, housing characteristics such as source of drinking water, and availability of toilet facilities. Geographic variables included place of residence (rural/urban), province and ecological zone (mountain/hill/terai). Province was categorized into seven administrative divisions, according to the current administrative structure of Nepal. Access to information included the frequency of reading newspapers, watching television and listening to the radio. A detailed study methodology and description of survey variables has been presented elsewhere^37^.

### Statistical analysis

The STATA version 14 (STATA corp) was used for analyses. We used sample weights to calculate national estimates from reported values. The association of explanatory and outcome variables was measured using Chi-square test. The multivariate logistic regression adjusting for all covariates was used to calculate adjusted odds ratio (AOR). The reference categories for calculation of adjusted odds ratios was selected on the basis of a research article from Nepal demographic health survey 2016^38^. The covariates (age, education, ecological zone, place of residence, wealth quintile and access to information) was entered simultaneously in the model. The cluster sampling design was adjusted in all the statistical analysis considering place of residence as a stratum and enumeration area as a cluster for this study. The geographic analysis of provinces of Nepal was performed in SAS v9.4 using gmap procedure^39^. We plotted graphs using all three DHS surveys data to visualize trend of tobacco use among Nepalese males and females. The existence of socio economic inequality in tobacco consumption was assessed by concentration curve. This curve has been used to measure and compare the degree of socio-economic status (SES) related inequality in various health indicators^40–42^. We plotted cumulative percentage of the sample, ranked by wealth quintile from lowest to highest on X-axis and the corresponding cumulative percentage of any form of tobacco use was plotted on Y-axis. For each of the three surveys, the percentage of the sample who lie within each quintile differs slightly from the nominal value, but the deviations are only of a few percent. A curve above the line of equality indicates a greater concentration of any form of tobacco use among the poor and a curve below the line indicates a greater concentration of tobacco use among the rich.

### Ethics Statement

The survey protocol was reviewed and approved by the Nepal Health Research Council (NHRC) and the ICF Institutional Review Board. Written informed consent was taken from the household head to carry out the interviews. Participants were informed about voluntary participation and confidentiality of information and could refrain from responding to any of the questions. It was ensured that the information provided by the participants or collected about the participants was protected and non-identifiable. All methods were performed in accordance with the relevant guidelines and regulations.

## Data Availability

The datasets generated during and/or analysed during the current study are available from the corresponding author on reasonable request.

## References

[1] World Health Organization: Global status report on noncommunicable diseases. Geneva: World Health Organization. (2014. Available online, http://www.who.int/nmh/publications/ncd-status-report-2014/en/ (accessed 6 May 2018)) (2014).

[2] Collaborators GBDT (2017). Smoking prevalence and attributable disease burden in 195 countries and territories, 1990–2015: a systematic analysis from the Global Burden of Disease Study 2015. Lancet.

[3] Tobacco killing in low-income and middle-income countries. *The Lancet* **379**, 1172 (2012). 10.1016/S0140-6736(12)60492-9 22464371

[4] World Health Organization Tobacco: Fact Sheet No. 339. (2015. Available online, http://www.who.int/mediacentre/factsheets/fs339/en/ (accessed 6 May 2018)).

[5] World Health Organization. WHO Framework Convention on Tobacco Control. World Health Organization; Geneva, Switzerland: 2003. (Available online, http://www.who.int/fctc/text_download/en/ (accessed on 6 May 2018)).

[6] Pradhan, P. M., Niraula, S. R., Ghimire, A., Singh, S. B. &Pokharel, P. K. Tobacco use and associated factors among adolescent students in Dharan, Eastern Nepal: a cross-sectional questionnaire survey. *BMJ Open* **3** (2013). 10.1136/bmjopen-2012-002123 PMC3585970 23418297

[7] World Health Organization Parties to the WHO Framework Convention on Tobacco Control. (Available online, http://www.who.int/fctc/signatories_parties/en/ (accessed on 6 May 2018)) (2015).

[8] American Cancer Society, Vital Strategies. The Tobacco Atlas Nepal. (Available online, http://tobaccoatlas.org/wp-content/uploads/pdf/nepal-country-facts.pdf (accessed 6 May 2018)).

[9] Khanal V, Adhikari M, Karki S (2013). Social determinants of tobacco consumption among Nepalese men: findings from Nepal Demographic and Health Survey 2011. Harm Reduct J.

[10] Sreeramareddy CT, Ramakrishnareddy N, Harsha Kumar H, Sathian B, Arokiasamy JT (2011). Prevalence, distribution and correlates of tobacco smoking and chewing in Nepal: a secondary data analysis of Nepal Demographic and Health Survey-2006. Subst Abuse Treat Prev Policy.

[11] World Health Organization report on the global tobacco epidemic, The MPOWER package. Geneva: World Health Organization. (2008. Available online, http://www.who.int/tobacco/mpower/2008/en/ (accessed 6 May 2018)) (2008).

[12] International Agency for Research on Cancer, Smokeless Tobacco and Some Tobacco-Specific N-Nitrosamines, vol. 89 of IARC Monographs on the Evaluation of Carcinogenic Risks to Humans, World Health Organization International Agency for Research on Cancer. (2007. Available online, https://monographs.iarc.fr/ENG/recentpub/mono89.pdf). PMC4781254 18335640

[13] Non communicable diseases risk factors: STEPS survey Nepal 2013. (Available online, http://www.searo.who.int/nepal/mediacentre/non_communicable_diseases_risk_factors_steps_survey_nepal_2013.pdf).

[14] Sreeramareddy CT, Pradhan PM, Mir IA, Sin S (2014). Smoking and smokeless tobacco use in nine South and Southeast Asian countries: prevalence estimates and social determinants from Demographic and Health Surveys. Popul Health Metr.

[15] Tsai YW, Tsai TI, Yang CL, Kuo KN (2008). Gender differences in smoking behaviors in an Asian population. J Womens Health (Larchmt).

[16] Sinha DN (2015). Trends of Smokeless Tobacco use among Adults (Aged 15–49 Years) in Bangladesh, India and Nepal. Asian Pacific Journal of Cancer Prevention.

[17] Ministry of Health and Population (MOHP) [Nepal], New ERA, and Macro International Inc. Nepal Demographic and Health Survey 2006. *Available online*, https://dhsprogram.com/pubs/pdf/FR191/FR191.pdf (2007).

[18] Ministry of Health and Population (MOHP) [Nepal], New ERA, and ICF International Inc. Nepal Demographic and Health Survey 2011. *Available online*, https://dhsprogram.com/pubs/pdf/fr257/fr257%5B13april2012%5D.pdf (2012).

[19] Ministry of Health [Nepal], New ERA, and ORC Macro. Nepal Demographic and Health Survey 2001. (Available online, https://dhsprogram.com/pubs/pdf/fr132/fr132.pdf) (2002).

[20] Government of Nepal and United Nations. Nepal Millennium Development Goals. Progress Report. Available from, https://odahioa.archive.knowledgearc.net/bitstream/handle/10642/4605/RESUBMISSION%20FINAL%20without%20TC.pdf?sequence=1&isAllowed=y (2013.)

[21] Hitchman SC, Fong GT (2011). Gender empowerment and female-to-male smoking prevalence ratios. Bull World Health Organ.

[22] Ministry of Health and Population (MOHP) [Nepal]. Brief profile on tobacco control in Nepal. *Available online*, http://www.who.int/fctc/reporting/party_reports/nepal_2012_annex2_tobacco_profile.pdf.

[23] SURYA NEPAL PRIVATE LIMITED. Report of the directors for the financial year ended 31st Asadh 2073 (15th July 2016) (Available online, http://www.itcportal.com/about-itc/shareholder-value/annual-reports/itc-annual-report-2017/pdf/Surya-Nepal-Private-Limited.pdf (accessed 6 May 2018)).

[24] Khan A (2014). Smokeless tobacco control policies in SouthAsia: a gap analysis and recommendations. Nicotine Tob Res.

[25] Tobacco control laws. Legislation by country, Nepal. (Available online, https://www.tobaccocontrollaws.org/legislation/country/nepal/pl-health-warnings (accessed 6 May 2018)).

[26] World Health Organization, South-East Asia Regional Office. Report of the expert group consultation on smokeless tobacco and public health in WHO-SEAR countries. New Delhi, India: World Health Organization, South-East Asia Regional Office. (2015. Available online, http://www.searo.who.int/tobacco/meetings/sea_tobacco_23_slt_mumbai_july_2015_report.pdf (accessed on 6th May 2018)).

[27] Karki, Y., Pant, K. & Pande, B. A Study on the Economics of Tobacco in Nepal. HNP Discussion Paper;. World Bank, Washington, DC. (2003. Available online, https://openknowledge.worldbank.org/handle/10986/13750 (accessed 6 May 2018)).

[28] Sinha DN, Gupta PC, Ray C, Singh PK (2012). Prevalence of smokeless tobacco use among adults in WHO South-EastAsia. Indian J Cancer.

[29] Singh A, Ladusingh L (2014). Prevalence and determinants of tobacco use in India: evidence from recent Global Adult Tobacco Survey data. PLoS One.

[30] Achia TN (2015). Tobacco use and mass media utilization in sub-Saharan Africa. PLoS One.

[31] Tafawa AO, Viswanath K, Kawachi I, Williams DR (2012). Mass media exposure, social stratification, and tobacco consumption among Nigerian adults. Cancer Causes Control.

[32] Sreeramareddy CT, Kishore P, Paudel J, Menezes RG (2008). Prevalence and correlates of tobacco use amongst junior collegiates in twin cities of western Nepal: a cross-sectional, questionnaire-based survey. BMC Public Health.

[33] Hurley SF, Matthews JP (2008). Cost-effectiveness of the Australian National Tobacco Campaign. Tob Control.

[34] Reed, H. The effects of increasing tobacco taxation: a cost benefit and public finances analysis. London, UK: Action on Smoking and Health. (2010).

[35] Ministry of Health and Population (MOHP) [Nepal]. Directive on printing warning messages and pictures on tobacco product boxes, packets, cartons, parcels and packaging materials. (2014. Available online, http://www.tobaccolabels.ca/wp/wp-content/uploads/2016/06/Nepal-2014-Directive-for-Printing-Warning-Message-and-Pictures-on-Tobacco-Product-Packaging-2014-English-translation.pdf (accessed 6 May 2018)).

[36] Plain packaging on the cards for tobacco products. In *The Kathmandu Post* (Kathmandu, Nepal, 2017. Available online, http://kathmandupost.ekantipur.com/news/2017-05-09/plain-packaging-on-the-cards-for-tobacco-products.html).

[37] Ministry of Health, Nepal; New ERA; and ICF. 2017. Nepal Demographic and Health Survey 2016. *Available online*, https://www.dhsprogram.com/pubs/pdf/fr336/fr336.pdf.

[38] Mehata S (2018). Prevalence, awareness, treatment and control of hypertension in Nepal: data from nationally representative population-based cross-sectional study. Journal of Hypertension.

[39] Jadoo, M. Geographic analysis with PROC GMAP. *Available online*, https://analytics.ncsu.edu/sesug/2016/RV-278_Final_PDF.pdf.

[40] Kakwani N, Wagstaff A, van Doorslaer E (1997). Socioeconomic inequalities in health: Measurement, computation, and statistical inference. Journal of Econometrics.

[41] O’Donnell, O.A. & Wagstaff, A. Analyzing health equity using household survey data: a guide to techniques and their implementation. *World Bank Publications* (2008).

[42] Wagstaff A, Van Doorslaer E (1994). Measuring inequalities in health in the presence of multiple-category morbidity indicators. Health economics.

[43] Mishra SR (2016). Current smokers’ perception of cigarette graphic health warnings and smoking habits: a cross sectional study from Nepal. Health Prospect: Journal of Public Health.

